# The Role of Cognitive Reserve in Protecting Cerebellar Volumes of Older Adults with mild Cognitive Impairment

**DOI:** 10.1007/s12311-024-01695-w

**Published:** 2024-04-19

**Authors:** Maria Devita, Giulia Debiasi, Mariagiulia Anglani, Chiara Ceolin, Ilaria Mazzonetto, Chiara Begliomini, Simone Cauzzo, Cecilia Raffaelli, Alessandro Lazzarin, Adele Ravelli, Alessandra Bordignon, Marina De Rui, Giuseppe Sergi, Alessandra Bertoldo, Daniela Mapelli, Alessandra Coin

**Affiliations:** 1 Department of General Psychology (DPG), University of Padua, Via Venezia 8, Padua, Italy; 2 Geriatrics Unit, Department of Medicine (DIMED), University of Padua, Via Giustiniani 2, Padua, Italy; 3 Department of Surgery, Oncology and Gastroenterology, University of Padua, Via Giustiniani 2, Padua, Italy; 4 Department of Information Engineering, University of Padua, Via Gardenigo 6/B, Padua, Italy; 5 Neuroradiology Unit, University Hospital of Padua, Via Giustiniani 2, Padua, Italy; 6 Department of Neurobiology, Care Sciences and Society, Karolinska Institutet and Stockholm University, Aging Research Center, Stockholm, Sweden; 7 Parkinson’s Disease and Movement Disorders Unit, Center for Rare Neurological Diseases, Department of Neurosciences, University of Padova, Via Belzoni 160, Padua, Italy; 8 Geriatrics Unit, Ospedale Fracastoro, Via Circonvallazione 1, San Bonifacio, Verona, Italy; 9 Neurology Unit, Ospedale San Bortolo, Viale Rodolfi 37, Vicenza, Italy; 10 Padova Neuroscience Center, University of Padua, Via Orus 2/B, Padua, Italy

**Keywords:** Cognitive reserve, Cerebellum, Cerebellar dysfunction, Cerebellar grey matter, Mild cognitive impairment

## Abstract

The present study aims to investigate the relationship between cerebellar volumes and cognitive reserve in individuals with Mild Cognitive Impairment (MCI). A description of proxies of cerebellar cognitive reserve in terms of different volumes across lobules is also provided. 36 individuals with MCI underwent neuropsychological (MoCA, MMSE, Clock test, CRIq) assessment and neuroimaging acquisition with magnetic resonance imaging at 3 T. Simple linear correlations were applied between cerebellar volumes and cognitive measures. Multiple linear regression models were then used to estimate standardized regression coefficients and 95% confidence intervals. Simple linear correlations between cerebellar lobules volumes and cognitive features highlighted a significant association between CRIq_Working activity and specific motor cerebellar volumes: Left_V (ρ = 0.40, p = 0.02), Right_V (r = 0.42, p = 0.002), Vermis_VIIIb (ρ = 0.47, p = 0.003), Left_X (ρ = -0.46, p = 0.002) and Vermis_X (r = 0.35, p = 0.03). Furthermore, CRIq_Working activity scores correlated with certain cerebellar lobules implicated in cognition: Left_Crus_II, Vermis VIIb, Left_IX. MMSE was associated only with the Right_VIIB volume (r = 0.35, p = 0.02), while Clock Drawing Test scores correlated with both Left_Crus_I and Right_Crus_I (r = -0.42 and r = 0.42, p = 0.02, respectively). This study suggests that a higher cognitive reserve is associated with specific cerebellar lobule volumes and that Working activity may play a predominant role in this association. These findings contribute to the understanding of the relationship between cerebellar volumes and cognitive reserve, highlighting the potential modulatory role of Working activity on cerebellum response to cognitive decline.

**Graphical Abstract:**

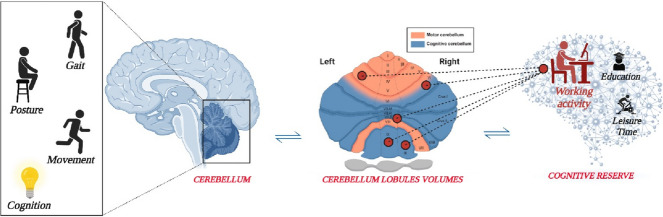

**Supplementary Information:**

The online version contains supplementary material available at 10.1007/s12311-024-01695-w.

## Introduction

For centuries, the cerebellum has been referred to control of movement, gait, and posture [1]. However, it has recently been acknowledged as playing an important role also in cognition, although how and to what degree remains an intriguing enigma [2]. A further missing piece concerns the possible involvement of the cerebellum in neurodegenerative disorders: if, on the one hand, it is considered immune to certain neurodegenerative processes [3], on the other hand the cerebellum seems to show structural and functional alterations associated with dementia [4, 5]. Direct investigation into reserve mechanisms has occurred within the brain, with numerous studies elucidating the connection between different reserve levels and structural or functional alterations in neurodegeneration. Conversely, research examining the correlation between reserve and the cerebellum is poor, and specifically, it has been explored to a lesser degree [6]. Cerebellar reserve, as well as cognitive reserve for the brain, has been referred to the capacity of the cerebellum to compensate for tissue damage or loss of function resulting from various etiologies. Owing to its remarkable plasticity, the cerebellum can adapt its structure and function in response to both beneficial and adverse conditions [7]. In scenarios of acute focal damage, Mitoma et al. [8] proposed that impaired cerebellar function could be compensated by other cerebellar regions or extracerebellar structures (i.e., structural cerebellar reserve). In contrast, when pathological changes gradually compromise cerebellar neuronal integrity, leading to cell death, it is possible that the affected area itself can compensate for the slowly evolving cerebellar lesion (i.e., functional cerebellar reserve). Accordingly, current evidence suggests that cerebellar reserve is enhanced by environmental enrichment through the mechanisms of autophagy and synaptogenesis, indicating that cerebellar reserve is not rigid or fixed but exhibits plasticity potentiated by experience [8, 9]. To the best of our knowledge, limited evidence exists on whether the entire cerebellar volume or specific lobules significantly correlate with high levels of cognitive reserve. This work aims to study the relationship between cerebellar volumes and cognitive reserve in a population of individuals with Mild Cognitive Impairment (MCI) through an innovative description of proxies of cerebellar cognitive reserve in terms of different volumes across lobules.

## Materials and methods

This observational study identified participants from those attending the outpatient clinic for Cognitive Decline and Dementia of the Geriatrics Unit, University Hospital of Padua. Individuals were recruited on a voluntary basis and after providing their informed consent. The project received approval from the Ethics Committee for Clinical Experimentation of the Province of Padua (code 5234/AO/21).

### Participants

*Inclusion Criteria:* diagnosis of Mild Cognitive Impairment (MCI); mild or mild-moderate dementia (last MMSE score between 18 and 24 inclusive); patients’ informed consent to participate to the study (even, if necessary, in the presence of a caregiver or appointed legal guardian—e.g., legal administrator if applicable-); psycho-physical ability to perform the required tasks.

*Exclusion Criteria:* diagnosis of advanced cognitive decline; severe sensorial deficits; presence of psychiatric disorders; poor quality of neuroimaging images.

Considering the above criteria, 40 subjects were enrolled, of which 4 were excluded due to poor quality of neuroimaging.

### Data Collection

*Informed Consent:* all participants were asked to sign an informed consent form to participate to the study and for processing personal data.

*Clinical Evaluation:* sociodemographic, clinical, and laboratory information were retrospectively obtained from medical records of outpatient visits at Centers for Cognitive Disorders and Dementia (CDCD). Information on remote, physiological, pharmacological, and social history.

*Neuroimaging:* patients underwent brain magnetic resonance imaging, as clinically indicated, at the Neuroradiology Unit—University Hospital of Padua. The examination was conducted according to a standardized protocol at the Center for individuals with cognitive decline.

### Assessment Tools

Cognitive Status Assessment:

- Mini Mental State Examination (MMSE; [10]): consists of 30 questions covering 7 cognitive areas (orientation in space and time, word registration, attention and calculation, recall, language, constructive praxis). The total score is adjusted based on the subject's age and education level and ranges from a minimum of 0 to a maximum of 30. A score equal to or less than 11 indicates severe cognitive decline, a score between 18 and 11 indicates moderate decline, a score between 18 and 24 indicates mild impairment, and a score between 24 and 30 indicates normal cognitive function.
- Montreal Cognitive Assessment (MoCA) [11]: a rapid screening tool validated for Mild Cognitive Impairment, consisting of 30 questions exploring various cognitive domains. The cognitive domains explored include attention, concentration, executive functions, memory, language, visuo-constructive skills, abstraction, calculation, and orientation. The maximum score is 30 points, with a score above 15.5 indicating a normal score.
- Clock Drawing Test (CDT, [12]): a cognitive test primarily assessing visuospatial and mental representation skills. The individual is presented with a sheet on which the clock face is already drawn, and he/she is required to place all the numbers in their correct positions. The individual is then asked to draw the clock hands to indicate a time communicated by the examiner. Scores are given from 0–4 for the presence of numbers, 0–3 for the arrangement of numbers, and 0–2.5 considering the presence and correct position of the clock hands; an additional 0.5 points are added if the hands differ in length. The maximum score is 10, and the normality cut-off depends on education and age group.
- Cognitive Reserve Assessment (Cognitive Reserve Index questionnaire -CRIq, [13]): after a brief collection of demographic data, the CRI-q proposes 20 items grouped into 3 sections, representing the main categories involved in the development of cognitive reserve throughout life: Education (years of school, attended extracurricular courses), Working activity (type of work done and for how many years), and Leisure time (activities performed, hobbies). The digital version of the questionnaire is free and available in multiple languages on the website http://cvi.math.unipd.it/CRIq. The CRI-q can be administered to the caregiver, family member, or the patient themselves; it can also be administered over the phone.

### Cerebellar Volume Measurement

Structural images of the cerebellum were obtained by means of magnetic resonance imaging (MRI) performed on a 3.0 T scanner (Ingenia, Philips Medical Systems, Best, Netherlands) equipped with a 32-channel head coil. High-resolution T1-weighted (T1w) images have been acquired using a 3D-TFE sequence with compressed sensing of 3.5, with TR = 6.7 ms, TE = 3.0 ms, flip angle = 8°, FOV = 240 × 240 mm^2^, and 1 mm isotropic resolution. N4 bias field correction [14] was applied on T1w images with the Advanced Normalization Tools (ANTs; [15]) from which also cerebellum and brain stem masks were derived and then used to keep only these anatomical structures, generating a masked-T1w. Spurious voxel remaining from the application of the masks were deleted with an ad-hoc code implemented in Matlab (Mathworks, Natick, MA, USA r2022a update 3 (9.12.0.1975300)). By means of the Spatially Unbiased Infra-Tentorial (SUIT; [16]) Matlab toolbox, the T1w image was used to determine the boundary box to isolate the cerebellum. The cropped volume obtained by applying the boundary box on the masked-T1w image was registered to the SUIT template in MNI space through affine and non-linear transformations using ANTs. The inverse transformation was then applied to the SUIT anatomical parcellation of the cerebellum to bring it into the subject’s T1w space. To verify the correct inclusion or exclusion of voxels from the cerebellum parcellations, these were visually inspected and manually corrected by an expert neuroradiologist where necessary, by using FSLeyes [17]. This was done by overlaying the parcellation of cerebellar lobules on the structural T1w. The SUIT toolbox, however, performs anatomical parcellation including also white matter, which should instead be excluded, as the volumes of interest pertain to gray matter. Therefore, individual white matter masks were created with FreeSurfer 7.1 ([18]; https://surfer.nmr.mgh.harvard.edu/). Brain extraction using ANTs was performed on the T1w image in order to estimate the affine transformation to the brain image obtained from FreeSurfer and then to apply it to the cerebellar parcellation. The white matter mask was then used to isolate grey matter tissue in the parcellation. Volumes of the cerebellar lobules were computed with an ad-hoc code written in Matlab.

### Statistical analyses

Characteristics of participants included in the study were expressed as means ± standard deviations for normally distributed quantitative variables and as medians and interquartile ranges for those with non-normal distributions. Normality of the distributions of quantitative variables was assessed using the Shapiro–Wilk test. Categorical variables were presented as frequencies and percentages. The sample was divided into two groups based on the median of the total cerebellar gray matter volume, considering it as the dividing value (GMCtv), after standardizing GMCtv for the participants' cerebral volume. The characteristics of participants with higher or lower GMCtv were analyzed and compared using Student's t-test and Mann–Whitney test for continuous variables, and Chi-square test and Fisher's exact test for categorical variables. To manage the risk of Type I error arising from multiple comparisons, we applied the Bonferroni correction. Simple linear correlations were applied between cerebellar volumes and variables of interest to study the correlation coefficient (Pearson's r or Spearman’s ρ, as appropriate). Multiple linear regression analysis with stepwise forward procedure was conducted to elucidate the relationship between cerebellar volumes and variables of interest, with a particular focus on cognitive reserve. The analysis aimed to estimate standardized regression coefficients (ß) and corresponding 95% confidence intervals (CI), allowing for a comprehensive exploration of the associations while controlling for potential confounders. The model was constructed to assess the impact of cerebellar volumes on cognitive reserve while adjusting for demographic and health-related variables. Initially, univariate regression models were employed to assess the correlation of patients' characteristics (age, gender, MMSE, CRIq scores, ADL scores, total number of comorbidities, educational level, social status) with cognitive reserve. Variables with a p-value < 0.20 were included in the multivariable model. In the multivariate model, the collinearity of the variables was assessed by the Variance Inflation Factor using a cut-off of 2 for exclusion. Standardized regression coefficients (ß) were computed to provide a measure of the strength and direction of the relationship between each predictor variable and cognitive reserve, ensuring comparability across predictors. The 95% confidence intervals (CI) for these coefficients were calculated to estimate the range within which the true population parameters are likely to lie, enhancing the robustness of the findings. Statistical tests were considered significant at p < 0.05. The analyses were performed using SPSS version 28.0.1.0 for Windows (IBM Corp., Armonk, NY).

## Results

Table 1 shows participants’ descriptive characteristics.

**Table 1:** Descriptive characteristics of the sample at baseline

Variables		Total (n = 36)
Age [years]		77.78 ± 4.34
Gender	*Male*	11 (30.6%)
	*Female*	25 (69.4%)
Education [years]		7.56 ± 3.52
Retirement age		60.47 ± 8.86
Comorbidities [number]		1.92 ± 1.78
Marital status	*Married*	14 (38.89%)
	*Widowed*	19 (52.78%)
	*Separated/divorced*	3 (8.33%)
Living situation	*With family*	20 (55.56%)
	*Family* + *home caregiver*	2 (5.56%)
	*Alone*	14 (38.89%)
Previous work experience	*Unskilled worker*	20 (55.56%)
	*Craftsman*	8 (22.22%)
	*Trader*	5 (13.89%)
	*Manager*	3 (8.33%)
Social isolation		12 (33.33%)
Cognitive characteristics	*MMSE*	24.58 ± 4.05
	*MOCA*	15.88 ± 5.25
	*Clock Drawing Test*	6.71 ± 3.59
	*CRIq*	98.14 ± 17.91

*Notes*: Values are expressed as mean ± standard deviation for continuous variables and as frequency (percentage) for dichotomous variables. *Abbreviations:* MMSE = Mini Mental State Examination; MOCA = Montreal Cognitive Assessment; CRIq = Cognitive Reserve Index questionnaire

Among the 36 participants, 25 (69.4%) were women, with an average age of 77.78 ± 4.34 years. The mean years of education and comorbidities stood at 7.56 ± 3.52 and 1.92 ± 1.78, respectively. Regarding living arrangements, 38.9% of participants were married, and more than half lived with their families (55.56%). A third of participants experienced social isolation. In terms of neuropsychological assessment, the MMSE and MOCA scores averaged 24.58 ± 4.05 and 15.88 ± 5.25, respectively, while the overall cognitive reserve averaged 98.14 ± 17.91.

Moreover, we tested the association between MOCA and CRIq scores, finding a significant positive association (R^2^ = 0.18, p = 0.02 – data not shown).

Table 2 presents the volumes of cerebellar lobules for the entire sample.

**Table 2:** Cerebellar lobes volumes in mm3 of study participants, entire cohort

MOTOR CEREBELLAR AREAS	Total (n = 36)	COGNITIVE CEREBELLAR AREAS	Total (n = 36)
*Left_I_IV*	*2146.64* ± *431.95*	***Left_Crus_I***	*11,604.33* ± *1516.17*
*Right_I_IV*	*2538.94* ± *563.66*	***Right_Crus_I***	*11,902.44* ± *1732.54*
*Left_V*	*3117.50* *(2695.25;3312.00)*	***Left_Crus_II***	*8255.86* ± *1240.69*
*Right_V*	*3369.31* ± *560.87*	***Vermis_Crus_II***	*464.56* ± *73.59*
*Left_VIIIa*	*4317.33* ± *548.15*	***Right_Crus_II***	*7876.08* ± *1139.61*
*Vermis_VIIIa*	*949.50* *(857.25;1053.75)*	***Left_VIIb***	*4104.00* *(3572.50;4511.50)*
*Right_VIIIa*	*4069.56* ± *561.96*	***Vermis_VIIb***	*181.86* ± *31.85*
*Left_VIIIb*	*3602.31* ± *395.79*	***Right_VIIb***	*4250.67* ± *625.57*
*Vermis_VIIIb*	*512.00* *(457.75;625.75)*	***Left_IX***	*2933.47* ± *411.56*
*Right_VIIIb*	*3559.81* ± *454.05*	***Vermis_IX***	*662.00* *(618.75;721.00)*
*Left_X*	*694.50* *(637.00;764.75)*	***Right_IX***	*3183.00* *(2971.50;3401.50)*
*Vermis_X*	*364.33* ± *70.41*		
*Right_X*	*652.58* ± *111.50*		
*COGNITIVE-MOTOR CEREBELLAR AREAS*		***TOTAL VOLUMES***	
*Left_VI*	*7428.58* ± *1005.42*	***GMCtv***	*101,464.22* ± *10,611.55*
*Vermis_VI*	*1724.06* ± *238.43*	***eTIV***	*1,414,266.42 (1,353,230.99;1,528,765.00)*
*Right_VI*	*6892.11* ± *1074.21*	***Vermis-tv***	*4930.81* ± *563.72*

*Notes*: Values are expressed as mean ± standard deviation or median (interquartile range) as appropriate

*Abbreviations*: GMCtv = Total gray matter cerebellum; eTIV = Estimated total intracranial volume; Vermis-tv: Vermis volume

In the group characterized by a lower GMCtv, the mean score obtained on the MMSE was 26.00 (interquartile range 23.75–28.25), while in the group with a higher volume of GMCtv the mean MMSE score was slightly lower, at 24.50 (interquartile range 18.75–28.25). However, this difference did not reach statistical significance (p = 0.31, corrected p-value = 0.17). Regarding the mean MOCA score, in the group with a lower volume of GMCtv, a mean value of 18.00 (interquartile range 13.50–22.00) was observed, while in the group with a higher volume of GMCtv, the mean score was 13.00 (interquartile range 10.00–19.00), and the difference was statistically significant (p = 0.05, corrected p-value = 0.04). The mean scores on the Clock Drawing Test, CRIq School, CRIq Work, CRIq Free Time, and CRIq Total did not show statistically significant differences between the two groups (please refer to Supplementary Table 1).

Upon examining the simple linear correlations between cerebellar lobules volumes and cognitive features, a notable association was found between CRIq_Working activity and specific motor cerebellar volumes [2]: Left_V (ρ = 0.40, p = 0.02), Right_V (r = 0.42, p = 0.002), Vermis_VIIIb (ρ = 0.47, p = 0.003), Left_X (ρ = -0.46, p = 0.002) and Vermis_X (r = 0.35, p = 0.03) (refer to Supplementary Table 2). Furthermore, CRIq_Working activity scores correlated with certain cerebellar lobules implicated in cognition [2]: Left Crus II, Vermis VIIb, Left_IX (see Supplementary Table 3). MMSE was associated only with the Right_VIIB volume (r = 0.35, p = 0.02), while Clock Drawing Test scores correlated with both Left_Crus_I and Right_Crus_I (r = -0.42 and r = 0.42, p = 0.02, respectively) (see Supplementary Table 3). Finally, CRIq_Working Activity correlated with nearly all cognitive-motor cerebellar volumes (refer to Supplementary Table 4).

Table 3 presents the results of multiple linear regression analysis between cerebellar volumes and cognitive predictors, adjusted for gender, age, comorbidity, and MMSE. This type of analysis was conducted on all cerebellar volumes. However, we chose to report only the significant data, both concerning the considered cerebellar volumes and the significantly associated covariates. Regarding motor cerebellar volumes, only Left_V was significantly associated with CRIq_Education (R^2^ = 0.45, p = 0.004); the volumes of Vermis VIIb and Vermis IX among cognitive volumes showed a significant association with CRIq_Working activity (R^2^ = 0.42, p = 0.01 and R^2^ = 0.51, p = 0.02, respectively). Concerning mixed cerebellar volumes (i.e., motor and cognitive), the highest correlations (see Supplementary Table 4) were between CRIq_Working Activity, CRIq_Education, and CRIq_Total score and the entire VI lobule. Finally, Vermis-tv was significantly associated with CRIq_Working Activity (R^2^ = 0.64, p = 0.005).

**Table 3:** Multiple linear regressions between cognitive cerebellar volumes and cognitive-motor cerebellar volumes and predictors of interest

	PREDICTORS	ADJUSTED TOTAL R^2^	BETA	P-VALUE
MOTOR CEREBELLAR VOLUMES				
LEFT_V	CRIq School	0.45	0.41	**0.004**
	CRIq Work	0.27	0.27	0.12
	CRIq Free Time	0.30	0.33	0.07
	CRIq Total	0.26	0.10	0.80
COGNITIVE CEREBELLAR VOLUMES				
VERMIS_VIIB	CRIq School	0.35	0.28	0.08
	CRIq Work	0.42	0.52	**0.001**
	CRIq Free Time	0.33	0.21	0.89
	CRIq Total	0.27	0.19	0.35
VERMIS_IX	CRIq School	0.35	0.12	0.96
	CRIq Work	0.51	0.48	**0.02**
	CRIq Free Time	0.27	0.22	0.15
	CRIq Total	0.26	0.21	0.33
RIGHT_IX	CRIq School	0.25	0.26	0.47
	CRIq Work	0.30	0.34	**0.03**
	CRIq Free Time	0.13	0.25	0.58
	CRIq Total	0.25	0.35	0.55
COGNITIVE-MOTOR CEREBELLAR VOLUMES				
RIGHT_VI	CRIq School CRIq Work	0.27 0.34	0.33 0.48	0.07 **0.01**
	CRIq Free Time	0.25	0.36	0.97
	CRIq Total	0.19	0.38	**0.04**
TOTAL CEREBELLAR VOLUMES				
VERMIS-TV	CRIq School CRIq Work	0.35 0.64	0.25 0.41	0.07 **0.005**
	CRIq Free Time	0.34	0.19	0.56
	CRIq Total	0.27	0.30	0.27

*Notes*: All analyses are adjusted for gender, age, comorbidity, and MMSE. *Abbreviations*: CRIq = Cognitive Reserve Index questionnaire; Vermis-tv: Vermis volume. Significant p-values are reported in bold

## Discussion

The present paper aimed to describe the relationship between cerebellar volumes and cognitive reserve in a population of individuals with MCI, with an innovative focus on how different proxies of cerebellar cognitive reserve correlate with lobules volumes, by means of advanced statistical neuroimaging analyses.

As it happens for the brain, also cerebellar volume may depend on how much the nervous system has been stimulated throughout lifespan [6, 8]. Indeed, it has been reported that sensory, motor, cognitive, and social stimulations significantly influence the structure and function of both the brain and cerebellum [19]. Cerebellar circuits exhibit significant neuroplastic properties, allowing the encoding of experiences and, consequently, to learn behaviors [7]. One of the main mechanisms of cerebellar plasticity modifies the density and dimensions of the dendritic spines of Purkinje cells, which receive all afferent information arriving at the cerebellar cortex and are, thus, adaptively controlled by environmental factors [20–22]. In this sense, environmental stimuli of different natures that individuals are exposed to throughout their lives could impact the cerebellum in both functional and structural terms, modifying the previously known concept of cerebral cognitive reserve and enriching it with a parallel cerebellar cognitive reserve that may play a modulatory role in the brain's response to cognitive decline. This is perfectly in line with one of the first results emerged in our study and that can seem apparently counterintuitive: MoCA scores occurred to be higher in individuals with lower cerebellar volume, compared to those with higher cerebellar volume. As a matter of facts, the theory of cognitive reserve has been proposed to account for the incongruence between the degree of brain/cerebellar damage or pathology (i.e. brain/cerebellar volume) and its clinical manifestations (i.e. performance at neuropsychological psychometric tests). This means that our participants with a more severe pathology show lower cerebellar volume but still can cope for cognitive tasks (MoCA), “masking” their real undergoing frailty.

The present study also revealed that specific lobules were associated with a higher cognitive reserve, and in particular with cognitive reserve_Working Activity and with cognitive reserve_Education. Areas tipically considered “motor” (Left V, Right V, Vermis VIIIa, Vermis X), “cognitive” (Left Crus I, Left VIIb, Vermis VIIb, Left IX, Vermis IX, Right IX), as well as “mixed” (Left VI, Vermis VI, Right VI) were positively correlated with cognitive reserve_Working Activity, highlighting that a higher cognitive reserve in this domain reflects a larger cerebellar volume. This is in line with the findings of Fallahpour et al., [23] who suggested that individuals engaged throughout their lives in cognitively demanding jobs have an increased cognitive reserve, that would allow them to effectively cope with cognitive decline associated with aging and delaying the onset of dementia [24]. Furthermore, the fact that the majority of cerebellar volume lobules, both motor and cognitive, as well as mixed, correlated with cognitive reserve_Working activity suggests that this domain is particularly relevant, if not predominant, for many individuals. This result may suggest a role of “compensation” of cognitive reserve relate to working activity that is reflected in a higher volume in specific areas of the cerebellum. It is possible that, to compensate for damage, cerebellum undergoes plastic rearrangements before succumbing to neurodegeneration [25]. The relationship between working activity and brain volume has been the subject of several studies. Research has shown that physical activity, including working activity, is associated with larger brain volumes in key areas. For example, a study found that regular engagement in physical activities such as walking, running, or sports was linked to larger brain volumes in total gray matter, white matter, hippocampus, and frontal, parietal, and occipital lobe [26]. Another study demonstrated that higher brain volumes in the parietal and temporal lobes, largely in white matter, are associated with higher daily physical activity in older adults [27]. These findings suggest that working activity, as a form of physical activity, may have a modulatory effect on brain volume. The characteristics of our sample may explain why cognitive reserve_working activity was found to be mostly associated with cerebellar lobules. The historical and social factors among current older adults may have played a role in shaping their leisure and work activities. In many countries, leisure activities, whether recreational, cultural, or social, were often accessible to only a few individuals, while the majority dedicated much of their time to work and family care. The sample of individuals under study mainly held jobs that were closely linked to motor activities, rather than purely cognitive ones. This pattern was influenced by social contextual characteristics that led people to seek employment to support their families and establish their own households, thus avoiding becoming a burden on their parents, who often had limited means. The societal and historical context, therefore, influenced the type of work and leisure activities in which older adults engaged [28, 29]. The component of cognitive reserve related to leisure time was not found to be significantly associated with cerebellar volumes, despite being an important determinant of cognitive reserve [28, 30, 31]. This may be because cerebellar atrophy is an indicator of previous cerebellar damage and thus responds to predictors that consider the entire span of life.

Some limitations should certainly be acknowledged: first of all, the lack of a control group. The results of the present study are limited to a group of individuals with mild cognitive impairment and cannot allow to make inferences on the influence that cerebellar cognitive reserve may have on potential clinical evolutions. For these reasons, this study should therefore be considered descriptive and further investigations will allow to apply these findings in the neurodegenerative field of study, also providing a comparison with healthy individuals. Secondly, the small sample size: the results showed should be considered carefully as potentially not representative of the entire population with MCI.

## Conclusions

This study suggests that a higher cognitive reserve, particularly in the working activity domain, correlates with larger volumes in certain cerebellar lobules. This association persists despite underlying neurodegenerative processes that may affect other areas of the cerebellum. Further investigations are needed in order to consolidate current findings and to suggest possible clinical applications that would allow to “exploit” cerebellar cognitive reserve for the care and cure of individuals with mild cognitive impairment.

## Supplementary Information

Below is the link to the electronic supplementary material.

Supplementary file1 (DOCX 49 KB)

## Data Availability

The data that support the findings of this study are not openly available due to reasons of sensitivity and are available from the corresponding author upon reasonable request. Data are located in controlled access data storage at University of Padua.
