# Supplementary material for: The Role of Cognitive Reserve in Protecting Cerebellar Volumes of Older Adults with mild Cognitive Impairment
Source: Cerebellum. 2024 Apr 19;23(5):1966–74. doi: 10.1007/s12311-024-01695-w (PMC11489282; doi:10.1007/s12311-024-01695-w)
Supplement: Supplementary file 1 — Supplementary file1 (DOCX 49 KB) [file 12311_2024_1695_MOESM1_ESM.docx]

**Supplementary Table 1.** **Descriptive cognitive characteristics of study participants stratified based on the median of GMCtv in mm^3^.**

| **VARIABLES** | **Minor GMCtv**  **(n=18)** | **Major GMCtv**  **(n=18)** | **p-value** | **Corrected p-value** |
| --- | --- | --- | --- | --- |
| **MMSE** | 26.00  (23.75;28.25) | 24.50  (18.75;28.25) | 0.31 | 0.17 |
| **MOCA** | 18.00  (13.50;22.00) | 13.00  (10.00;19.00) | **0.05** | **0.04** |
| **Clock Drawing Test** | 7.00  (3.00;10.00) | 8.50  (4.75;10.00) | 0.84 | 0.60 |
| **CRIq School** | 96.00  (90.50;102.75) | 93.00  (89.00;101.25) | 0.50 | 0.37 |
| **CRIq Work** | 89.00  (78.75;111.00) | 83.50  (73.75;97.25) | 0.18 | 0.21 |
| **CRIq Free Time** | 102.50  (89.25;122.75) | 111.50  (91.00;117.00) | 0.73 | 0.77 |
| **CRIq Total** | 100.00  (87.00;108.75) | 95.00  (84.25;110.00) | 0.58 | 0.39 |

### Notes: Values are expressed as median (interquartile range).

### *Abbreviations*: MMSE = Mini Mental State Examination; MOCA = Montreal Cognitive Assessment; CRIq = Cognitive Reserve Index questionnaire; GMCtv = Total gray matter cerebellum.

**Supplementary Table 2. Simple linear correlations between motor cerebellar volumes and cognitive features.**

###
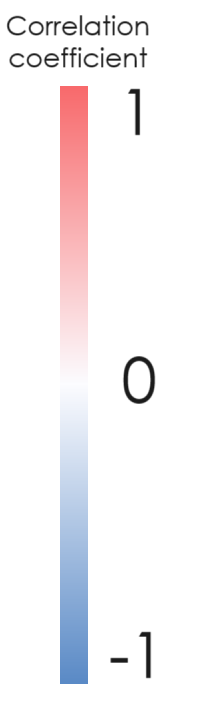


|  | **Criq School** | **Criq Work** | **Criq Free-Time** | **Criq Total** | **MMSE** | **MOCA** | **CDT** |
| --- | --- | --- | --- | --- | --- | --- | --- |
| **Left_I_IV** |  |  |  |  |  |  |  |
| **Right_I_IV** |  |  | **X** |  |  |  |  |
| **Left_V** |  | **X** |  |  |  |  |  |
| **Right_V** |  | **X** |  |  |  |  |  |
| **Left_VIIIa** |  |  |  |  |  |  |  |
| **Vermis_VIIIa** |  |  |  |  |  |  |  |
| **Right_VIIIa** |  |  |  |  |  |  |  |
| **Left_VIIIb** |  |  |  |  |  |  |  |
| **Vermis_VIIIb** |  | **X** |  |  |  |  |  |
| **Right_VIIIb** |  |  |  |  |  |  |  |
| **Left_X** |  | **X** |  |  |  |  |  |
| **Vermis_X** |  | **X** |  |  |  |  |  |
| **Right_X** |  |  |  |  |  |  |  |

*Abbreviations*: CRIq = Cognitive Reserve Index questionnaire; MMSE = Mini Mental State Examination; MOCA = Montreal Cognitive Assessment; CDT = Clock Drawing Test. X means significant correlations (p<0.05).

**Supplementary Table 3. Simple linear correlations between cognitive cerebellar volumes and cognitive features.**


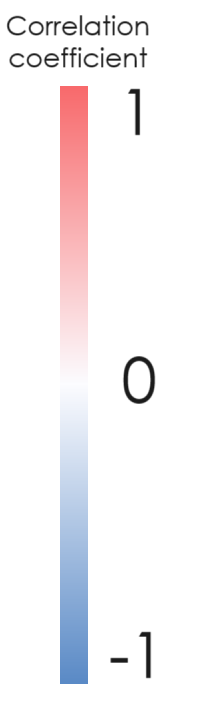


|  | **Criq School** | **Criq Work** | **Criq Free-Time** | **Criq Total** | **MMSE** | **MOCA** | **CDT** |
| --- | --- | --- | --- | --- | --- | --- | --- |
| **Left_Crus_I** |  |  |  |  |  |  | **X** |
| **Right_Crus_I** |  |  |  |  |  |  | **X** |
| **Left_Crus_II** |  | **X** |  |  |  |  |  |
| **Vermis_Crus_II** |  |  |  |  |  |  |  |
| **Right_Crus_II** |  |  |  |  |  |  |  |
| **Left_VIIb** |  |  |  |  |  |  |  |
| **Vermis_VIIb** |  | **X** |  |  |  |  |  |
| **Right_VIIb** |  |  |  |  | **X** |  |  |
| **Left_IX** |  | **X** |  |  |  |  |  |
| **Vermis_IX** |  |  |  |  |  |  |  |
| **Right_IX** |  |  |  |  |  |  |  |

*Abbreviations:* CRIq = Cognitive Reserve Index questionnaire; MMSE = Mini Mental State Examination; MOCA = Montreal Cognitive Assessment; CDT = Clock Drawing Test. X means significant correlations (p<0.05).

**Supplementary Table 4. Simple linear correlations between cognitive-motor cerebellar volumes and cognitive features.**

###
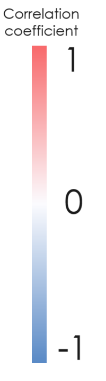


|  | **Criq School** | **Criq Work** | **Criq Free-Time** | **Criq Total** | **MMSE** | **MOCA** | **CDT** |
| --- | --- | --- | --- | --- | --- | --- | --- |
| **Left_VI** |  | **X** |  |  |  |  |  |
| **Vermis_VI** |  | **X** |  |  |  | **X** |  |
| **Right_VI** |  |  |  |  |  |  |  |
| ***Total Cerebellar Volumes*** |  |  |  |  |  |  |  |
| **eTIV** |  |  |  |  |  |  |  |
| **Vermis-tv** |  | **X** |  |  |  | **X** |  |

### *Abbreviations*: CRIq = Cognitive Reserve Index questionnaire; MMSE = Mini Mental State Examination; MOCA = Montreal Cognitive Assessment; CDT = Clock Drawing Test; eTIV = Estimated total intracranial volume; Vermis-tv: Vermis volume. X means significant correlations (p<0.05).
